# Enhanced expression of IFN- **γ** mRNA in CD4^+^or CD8^+^tumour-infiltrating lymphocytes compared to peripheral lymphocytes in patients with renal cell cancer

**DOI:** 10.1054/bjoc.2000.1275

**Published:** 2000-08-16

**Authors:** U Elsässer-Beile, M Rindsfüser, T Grussenmeyer, W Schultze-Seemann, U Wetterauer

**Affiliations:** Department of Urology, University of Freiburg, Experimental Research Group, Stefan Meier Strasse 8, Freiburg, D-79106, Germany

**Keywords:** renal cell carcinoma, TIL, cytokines, Fas ligand

## Abstract

The mRNA expression of the cytokines IFN-γ, IL-10 and TNF-α and the proapoptotic factor Fas ligand (FasL) was compared in freshly isolated CD4^+^and CD8^+^tumour-infiltrating lymphocytes (TIL) and simultaneously obtained autologous CD4^+^and CD8^+^peripheral blood lymphocytes (PBL) from 20 patients with renal cell carcinomas (RCC). TIL were isolated from mechanically disaggregated tumour material and PBL from peripheral blood by gradient centrifugation. The cells of the interphase were depleted from tumour cells with anti-human epithelial antigen magnetic beads and then positive selection was performed with anti-CD4 or anti-CD8 magnetic beads. In these pure lymphocyte preparations the constitutive expression of cytokine and FasL mRNAs was determined by using a PCR-assisted mRNA amplification assay. In the CD4^+^TIL from the 20 patients with RCC, levels of mRNAs encoding for IFN-γ (*P*≤ 0.001), IL-10 (*P*≤ 0.05), and FasL (*P*≤ 0.001) were significantly higher than in the autologous CD4^+^PBL. Comparison of CD8^+^TIL and CD8^+^PBL revealed a significant higher expression of IFN-γ (*P*≤ 0.001), IL-10 (*P*≤ 0.01) and FasL mRNAs (*P*≤ 0.001) in the former. However, TNF-α mRNA levels were significantly lower in the CD8^+^TIL than in the CD8^+^PBL (*P*≤ 0.05). These data reflect a general in vivo activation of RCC infiltrating lymphocytes in the tumour surrounding. © 2000 Cancer Research Campaign


*British Journal of Cancer* (2000) **83**(5), 637–641
© 2000 Cancer Research Campaign
doi: 10.1054/ bjoc.2000.1275, available online at http://www.idealibrary.com on IDEAL®

### Enhanced expression of IFN-γ mRNA in CD4⁺ or CD8⁺ tumour-infiltrating lymphocytes compared to peripheral lymphocytes in patients with renal cell cancer

**U Elsässer-Beile, M Rindsfüser, T Grussenmeyer, W Schultze-Seemann and U Wetterauer**

Department of Urology, University of Freiburg, Experimental Research Group, Stefan Meier Strasse 8, D-79106 Freiburg, Germany

**Summary** The mRNA expression of the cytokines IFN-γ, IL-10 and TNF-α and the proapoptotic factor Fas ligand (FasL) was compared in freshly isolated $CD4^+$ and $CD8^+$ tumour-infiltrating lymphocytes (TIL) and simultaneously obtained autologous $CD4^+$ and $CD8^+$ peripheral blood lymphocytes (PBL) from 20 patients with renal cell carcinomas (RCC). TIL were isolated from mechanically disaggregated tumour material and PBL from peripheral blood by gradient centrifugation. The cells of the interphase were depleted from tumour cells with anti-human epithelial antigen magnetic beads and then positive selection was performed with anti-CD4 or anti-CD8 magnetic beads. In these pure lymphocyte preparations the constitutive expression of cytokine and FasL mRNAs was determined by using a PCR-assisted mRNA amplification assay. In the $CD4^+$ TIL from the 20 patients with RCC, levels of mRNAs encoding for IFN-γ ($P \leq 0.001$), IL-10 ($P \leq 0.05$), and FasL ($P \leq 0.001$) were significantly higher than in the autologous $CD4^+$ PBL. Comparison of $CD8^+$ TIL and $CD8^+$ PBL revealed a significant higher expression of IFN-γ ($P \leq 0.001$), IL-10 ($P \leq 0.01$) and FasL mRNAs ($P \leq 0.001$) in the former. However, TNF-α mRNA levels were significantly lower in the $CD8^+$ TIL than in the $CD8^+$ PBL ($P \leq 0.05$). These data reflect a general in vivo activation of RCC infiltrating lymphocytes in the tumour surrounding. © 2000 Cancer Research Campaign

**Keywords**: renal cell carcinoma; TIL; cytokines; Fas ligand

The implication of T-cell-mediated immunity in renal cell carcinoma (RCC) was suggested when lymphocyte-rich infiltrates, mainly composed of $CD3^+$ T cells, have been found in tumours (Mitropoulos et al, 1994). Additionally, it has been shown that some of the tumour-infiltrating lymphocyte (TIL) clones can specifically, recognize and respond to autologous tumour cells (Finke et al, 1992; 1994; Schendel et al, 1993; Brouwenstijn et al, 1996; Caignard et al, 1996). However, freshly isolated TIL in bulk cultures were found partially or completely unable to exhibit cytotoxic activity as compared to normal T cells, i.e. they seem to be compromised in their antitumour activity (Whiteside and Parmiani, 1994; van den Hove et al, 1997).

Local cytokine production is thought to play a central regulatory role in the activation of infiltrating lymphocytes and may have an impact on the development of an effective antitumour response. It has been shown that also expression of Fas ligand (FasL) is induced during T cell stimulation and, therefore, may serve as activation marker (Berken, 1995).

Because little is known about the immunological activation status of RCC TIL in the tumour environment, the aim of the present study was to characterize freshly isolated pure $CD4^+$ and $CD8^+$ TIL from RCC with respect to the expression of Th1-type and Th2-type cytokine and FasL mRNAs as compared to autologous peripheral lymphocytes.

*Received 2 February 2000*
*Revised 5 April 2000*
*Accepted 10 April 2000*

*Correspondence to:* U. Elsässer-Beile

### MATERIALS AND METHODS

### Tumour and blood samples

Tumour tissue (2–6 g) and heparinized peripheral blood samples (8 ml) were obtained from 20 patients (12 men and 8 women) between ages 32 and 84 years with histologically verified renal cell carcinomas undergoing therapeutic surgery. The histopathology of the 20 tumours was a clear-cell adenocarcinoma. Pathological stages included pT1 ($n$ = 8), pT2b ($n$ = 4), pT3a ($n$ = 2), and pT3b ($n$ = 6). The grading of the tumours was G1 ($n$ = 1), G2 ($n$ = 17) or G3 ($n$ = 2). Blood was taken at the onset of surgery. Separation procedures applied to blood and tumour samples were started within 30 min after the operation. None of these patients had received preoperative antitumour therapy.

### Isolation of $CD4^+$ and $CD8^+$ TIL and PBL

After removing necrotic areas and fat, the tumour specimens were washed in phosphate buffered saline (PBS), minced to small pieces, washed again in PBS and then gently homogenized in a ‘loose-fitting’ hand homogenizer. In order to avoid all aggressive methods and long preparation times no enzymatic digestion was applied. The resulting cell suspension was passed over a 30 μm nylon mesh filter, overlaid onto Ficoll-Paque (Pharmacia) and centrifuged at 400 ***g***. The cells of the interphase were collected and washed twice. This crude lymphocyte preparation was depleted of adherent epithelial cells by using antihuman epithelial antigen microbeads (Miltenyi Biotec, Bergisch Gladbach, Germany). Then the sample was divided into two parts, from one of which $CD4^+$ and from the other $CD8^+$ lymphocytes were selected using magnetic beads directed against these determinants (Miltenyi). Incubation times with the magnetic beads were only 15 min.

PBL were isolated from heparinized blood by Ficoll density centrifugation. Mononuclear cells were collected from the interphase and washed twice. From these preparations $CD4^+$ and $CD8^+$ cell fractions were selected with magnetic beads.

All cells were counted, and $10^4$–$10^5$ cells were lysed in 350 µl of a solution consisting of 4 M guanidine thiocyanate, 25 mM sodium citrate, 0.5% sodium N-laurosylsarcosinate and 100 mM 2-mercaptoethanol.

### Quantification of cytokine mRNA expression by RT-PCR

Total RNA was isolated with silicagel-based membranes (Rneasy, Qiagen, Hilden, Germany) according to the manufacturer's protocol. cDNA synthesis was performed at 42°C for 60 min in a final volume of 50 µl which contained 25 µl of denatured RNA, 10 µl 5× buffer (Promega, Heidelberg, Germany), 5 µl of 10 mM dNTP (dATP, dCTP, dGTP, dTTP, Promega), 1.5 µl RNAsin (40 units $\mu l^{-1}$, Promega) 2.5 µl of 150 pM random hexamer primers, and 2.5 µl of AMV reverse transcriptase (10 units $\mu l^{-1}$, Promega).

Samples were subsequently diluted in 1:4 steps and 2 µl were combined with the PCR mixture, containing 11 µl water, 2 µl 10× buffer (Promega), 2 µl of 10 mM dNTP, 1 µl of 25 pM sense- and antisense-primer and 1 µl (1 U) Taq polymerase (Promega). For all cytokines, FasL, and β-actin cDNAs, internal standards had been constructed that were added to the PCR reactions at a constant low amount (unpublished data).

The reaction mixture was amplified with a thermal cycler (Mastercycler, Eppendorf, Hamburg, Germany) for 33 cycles. To exclude that contaminating genomic DNA might give interfering misleading signals, each lymphocyte RNA preparation was tested with each primer set also directly without reverse transcription. Prior to testing for the presence of cytokine-related transcripts, a PCR using a primer set to amplify β-actin cDNA was run at six dilutions together with a β-actin fragment (254 bp) of defined concentration in order to prove the integrity of the extracted RNA and to standardize the template. Because it is known that a number of processed β-actin pseudogenes exists (Ng et al, 1985; Raff et al, 1997), a primer set 5′-ACGGCTGCTTCCAGCTCCTC-3′ and 5′-TTGTTTTCTGCGCAAGTTAG-3′, (62°C) was chosen that had been proven not to amplify pseudogenes in control experiments without reverse transcription.

To exclude contamination with tumour cells, each TIL RNA preparation with tested with a primer set detecting transcripts of the human carbonic anhydrase XII gene that is expressed in renal cancer cells (Tureci et al, 1998). With the primer set 5′-CCGCCGAGCTGCACATTGTC-3′ and 5′-GGGCCAGTGAGAGGATGATG-3′ we found specific signals in all RCC tumour samples tested, but not in lymphocytes. Mixing experiments showed that a tumour-cell contamination in lymphocytes of less than 1% could be easily detected with 33 cycles. Only TIL samples without detectable tumour cell contamination were used for further analysis.

The following oligonucleotide 5′ and 3′ primer sequences were used for the PCR analysis of the cytokine and FasL mRNAs (annealing temperatures in brackets): IL-10 mRNA: 5′-ATGGGGGTTGAGGTATCAGAGG-3′ and 5′-ATGCCCCAAGCTGAGAACCAAG-3′ (64°C); IFN-γ mRNA: 5′-ACCGAATAATTAGTCAGCTT-3′ and 5′-AGTTATATCTTGGCTTTTCA-3′ (54°C); TNF-α mRNA: 5′-GGGGTACCTGGAAAGGACACCATAGA-3′ and 5′-GCTCTAGACCTTGGTCTGGTAGGA-3′ (58°C); FasL mRNA: 5′-GGATTGGGCCTGGGGATGTTTCA-3′ and 5′-TTGTGGCTCAGGGGCAGGTTGTTG-3′ (64°C).

PCR products were separated on 6% polyacrylamide gels and detected by ethidium bromide staining. Gel bands were measured densitometrically and pixel intensities of those bands which were in the linear range and best comparable for TIL and PBL were evaluated and multiplied with the dilution factor. Identification of the amplification products was done with restriction endonuclease analysis, sequence analysis and size determination. Sizes of the amplified fragments were as follows: IL-10 mRNA: 410 bp; IFN-γ mRNA: 357 bp; TNF-α mRNA: 527 bp; FasL mRNA 344 bp.

### Statistical methods

The relations between mRNA expression (pixel intensity of the PCR bands multiplied with the dilution) in the corresponding $CD4^+$ TIL and $CD4^+$ PBL as well as in the autologous $CD8^+$ TIL/$CD8^+$ PBL pairs were statistically evaluated using the paired Wilcoxon test.

### RESULTS

### Expression of mRNA for cytokines and FasL in $CD4^+$ TIL compared to autologous $CD4^+$ PBL and in $CD8^+$ TIL compared to $CD8^+$ PBL

From 5 g of tumour material the recovery of $CD4^+$ TIL and $CD8^+$ TIL was between $5 \times 10^4$ and $8 \times 10^5$ cells. In order to avoid long in vitro incubation times that may affect the activation status of the lymphocytes, no enzymatic digestion was applied, which may explain the relatively low recovery of TIL.

Prior to the analysis of cytokine gene expression by PCR-assisted mRNA amplification in TIL and PBL extracts, the samples were standardized for β-actin mRNA levels by a PCR at six 1:4 dilutions together with a competitor-β-actin fragment of defined concentration, so that equal amounts of β-actin cDNA template were used in the corresponding autologous $CD4^+$ TIL/PBL-pairs and $CD8^+$ TIL/PBL-pairs. Additionally, for each TIL RNA sample a PCR detecting human carbonic anhydrase XII mRNA was run to exclude a contamination with tumour cells.

PCR experiments were run in multiple dilutions to semi-quantitate the expression of the cytokine and FasL mRNAs. Corresponding PCR bands of $CD4^+$ TIL and autologous $CD4^+$ PBL, as well as $CD8^+$ TIL and autologous $CD8^+$ PBL, which were always derived from the same experiment, were quantified densitometrically and compared.

In the $CD4^+$ TIL from the 20 patients with RCC, levels of mRNAs encoding for IFN-γ ($P \le 0.001$), IL-10 ($P \le 0.05$), and FasL ($P \le 0.001$) were significantly higher than in the autologous $CD4^+$ PBL. Comparison of $CD8^+$ TIL and $CD8^+$ PBL of these patients revealed a significant higher expression of IFN-γ ($P \le 0.001$), IL-10 ($P \le 0.01$) and FasL mRNAs ($P \le 0.001$) in the former. However, TNF-α mRNA levels were significantly lower in the $CD8^+$ TIL than in the $CD8^+$ PBL ($P \le 0.05$). All data are summarized in Table 1.

In order to prove that the somewhat different method used for the purification of the TIL, including mechanic disaggregation of

© 2000 Cancer Research Campaign

**Table 1** Relative mRNA levels of cytokines in freshly isolated $CD4^+$ and $CD8^+$ TIL and simultaneously obtained autologous PBL from 20 RCC patients

| Patient | IFN-γ | | | | IL-10 | | | | TNF-α | | | | FasL | | | |
|---|---|---|---|---|---|---|---|---|---|---|---|---|---|---|---|---|
| | CD4+ | | CD8+ | | CD4+ | | CD8+ | | CD4+ | | CD8+ | | CD4+ | | CD8+ | |
| No. | TIL | PBL | TIL | PBL | TIL | PBL | TIL | PBL | TIL | PBL | TIL | PBL | TIL | PBL | TIL | PBL |
| 1 | **890** | 2 | **7100** | 2 | **11 000** | 1100 | **7200** | 860 | **3500** | 630 | **2600** | 190 | **590** | 10 | **2600** | 1200 |
| 2 | **170** | 1 | **12** | 2 | **730** | 10 | **400** | 21 | **6600** | 1000 | 550 | **1500** | **54** | 6 | **5100** | 940 |
| 3 | **7** | 2 | **2** | 0 | **150** | 81 | **120** | 71 | **1700** | 440 | **6700** | 1400 | **330** | 26 | **4600** | 370 |
| 4 | **2900** | 100 | **21 000** | 660 | **320** | 82 | **29** | 6 | 1000 | **3200** | 970 | **2700** | **490** | 28 | **1300** | 300 |
| 5 | **440** | 48 | **2100** | 55 | **600** | 120 | **900** | 79 | **120** | 100 | 150 | **420** | **65** | 48 | **530** | 120 |
| 6 | **140** | 4 | **310** | 68 | **75** | 9 | 16 | 16 | **260** | 160 | 230 | **300** | **490** | 28 | 810 | **1700** |
| 11 | **1500** | 350 | **6000** | 440 | **200** | 110 | **450** | 21 | **580** | 160 | 360 | **2900** | 330 | **2200** | **900** | 410 |
| 12 | **2900** | 430 | **24 000** | 4800 | **67** | 42 | 5 | **61** | 650 | **3000** | 470 | **3500** | **400** | 7 | 510 | **2800** |
| 13 | **1700** | 510 | **7500** | 760 | **180** | 81 | **58** | 14 | 1500 | **1700** | **1700** | 1200 | **690** | 78 | **17 000** | 1800 |
| 14 | **1100** | 4 | **6400** | 140 | **290** | 88 | 46 | **64** | 190 | **1800** | 480 | **4700** | **600** | 61 | **4000** | 340 |
| 15 | **890** | 340 | **2000** | 1600 | **280** | 180 | **740** | 140 | 1800 | **3900** | **5000** | 3300 | **610** | 85 | **4200** | 1900 |
| 16 | 480 | **1300** | 16 | **140** | **1500** | 550 | **230** | 160 | **1100** | 810 | 330 | **560** | 310 | **2100** | 34 | **59** |
| 17 | 240 | **320** | **8700** | 560 | **85** | 56 | **110** | 85 | 690 | **1000** | 93 | **900** | **1600** | 26 | **2300** | 970 |
| 18 | **820** | 170 | **2100** | 260 | **420** | 190 | 22 | **27** | 730 | **1400** | 860 | **1600** | **240** | 52 | **310** | 120 |
| 19 | **710** | 160 | **3100** | 670 | 670 | **900** | 410 | **590** | 950 | **2000** | **4400** | 2100 | **410** | 140 | **5600** | 270 |
| 21 | **230** | 45 | **1000** | 450 | 420 | **640** | **2600** | 350 | **5300** | 1600 | 380 | **2100** | **690** | 200 | **94** | 1 |
| 22 | **530** | 230 | **4500** | 530 | **260** | 120 | **340** | 110 | 63 | **1700** | 620 | **2300** | **260** | 24 | **5600** | 200 |
| 23 | **940** | 920 | 940 | **1300** | 1800 | **2900** | 540 | **1500** | **16 000** | 11 000 | 4600 | **13 000** | 1300 | 1300 | 2200 | **3300** |
| 24 | **470** | 360 | **2300** | 700 | **630** | 190 | **240** | 73 | **3800** | 2300 | 1200 | **3000** | 290 | **400** | **7000** | 78 |
| 25 | **170** | 57 | **5700** | 220 | 4 | **45** | **55** | 4 | 240 | **1300** | 440 | **1200** | **2300** | 150 | **8800** | 97 |

cDNA of each TIL/PBL pair was standardized for β-actin cDNA levels. Gel bands were measured densitometrically and pixel intensities were corrected for the dilution. For each TIL/PBL combination the higher number is highlighted by bold face the tumour material, does not induce a measurable cytokine expression in lymphocytes, in some experiments PBL were also treated in the homogenizer. In these experiments it could be shown that this mechanical treatment did not induce cytokine expression. Additionally, by comparison of $CD4^+$ PBL obtained by depletion or by positive selection, it could be demonstrated that the short incubation time of 15 min with magnetic beads does not affect cytokine mRNA expression in these cells (data not shown).

### Comparison of mRNA expression for cytokines and FasL in $CD4^+$ TIL and simultaneously obtained autologous $CD8^+$ TIL

Comparison of $CD4^+$ TIL and simultaneously obtained autologous $CD8^+$ TIL revealed a significant higher expression of IFN-γ mRNA ($P \leq 0.05$) and FasL mRNA ($P \leq 0.01$) in the $CD8^+$ TIL. Expression of IL-10 and TNF-α mRNA was not significantly different in both populations.

### DISCUSSION

One of the key functional parameters of an immune response is the local production of cytokines. There are, however, considerable problems involved in the characterization of the cytokine profile of TIL. These include the limited number of available TIL, difficulties in getting a population free of tumour cells and possible effects of the isolation procedure on cytokine production.

To overcome some of these problems we have investigated the constitutive expression of cytokine and FasL mRNA in pure $CD4^+$ and $CD8^+$ TIL which were freshly isolated by gradient centrifugation and selection with magnetic beads. Whereas TIL preparations separated from tumour material by gradient centrifugation alone were reported to contain 10–95% lymphocytes (Whiteside et al, 1986) and 6–75% tumour cells (Belldegrun et al, 1988) the isolation method with anti-human epithelial antigen and anti-CD4 or anti-CD8 magnetic beads used in the present study resulted in highly enriched TIL populations which were virtually free from epithelial cells.

We found a significant higher expression of IFN-γ, IL-10, and FasL mRNA in the isolated $CD4^+$ TIL and $CD8^+$ TIL populations as compared to autologous peripheral lymphocytes This may reflect an immunological activation of the TIL in the tumour environment. It may be concluded from the concomitantly high levels of IL-10 mRNA and IFN-γ mRNA in $CD8^+$ TIL that Th1 as well as Th2 lymphocytes are activated, which is in accordance with a recently published study showing also high levels of IL-10 and IFN-γ mRNA in TIL from non-small cell lung cancer and ovarian cancer biopsies (Asselin-Paturel et al, 1998; Pisa et al, 1992). Since it has recently been shown that in vitro activated T cells express high levels of FasL mRNA and protein (Alderson et al, 1995; Brunner et al, 1995; Dhein et al, 1995; Martinez-Lorenzo et al, 1996), the elevated FasL mRNA levels in RCC TIL also indicate an immunological activation of these lymphocytes.

There are only few studies on cytokine mRNA levels in TIL from RCC in the literature. Our finding of high IL-10 mRNA levels in TIL is in accordance with results reported by Wang et al, who detected IL-10 mRNA in freshly-isolated lymphocyte-enriched preparations from 4/5 RCC tumour specimens (Wang et al, 1995) and by Maeurer et al, who found that uncultured TIL from seven RCC patients expressed IL-10 and IL-4 mRNA (Maeurer et al, 1995). Other authors reported a high expression of IL-10 mRNA in biopsies of renal cell carcinomas (Filgueira et al, 1993; Nakagomi et al, 1995; Olive et al, 1997). A concomitant high expression of the Th2 cytokine IL-10 and the Th1 cytokine IFN-γ has been recently shown in isolated $CD3^+$ TIL from RCC (Elsässer-Beile et al, 1999).

*© 2000 Cancer Research Campaign*

IFN-γ is thought to play an important role in tumour lysis by T-cells, since it was shown that $CD8^+$ TIL lines from RCC that were specifically lytic also produced IFN-γ in response to autologous RCC cells but not allogeneic RCC cells (Finke et al, 1994). Recently, it was demonstrated in a syngeneic mouse model that TIL stimulated in vitro with tumour cells from the tumour of origin secreted relatively high levels of IFN-γ (Nagoshi et al, 1998; 1999). Therefore, high level expression of IFN-γ mRNA in $CD4^+$ and $CD8^+$ TIL from RCC reported here indicates a tumour-specific activation of these cells in vivo.

However, a high IFN-γ mRNA expression in the TIL may not necessarily indicate that this cytokine is also produced and secreted, which is expected to be necessary for an antitumour response.

Unfortunately, detection of cytokine production in lymphocytes at the protein level by FACS analysis only gives reproducibly measurable values after in vitro stimulation. This has been shown in numerous studies. There are two recent published studies measuring intracellular cytokines in TIL from lung carcinomas. Ito et al (1999) used a crude lymphocyte preparation stimulated in vitro with PMA and ionomycin. Ortegel et al (2000) compared stimulated and unstimulated TIL and found that in the absence of activation, cytokines could be detected only in less than 4% of the $CD3^+$ TIL.

Whereas isolated TIL have been shown to have a normal capacity to produce cytokines in vitro upon stimulation with anti-CD3 antibodies or mitogens (Elsässer-Beile et al, 1996; Angevin et al, 1997) cytotoxicity data obtained with freshly isolated $CD8^+$ TIL suggest that these cells may not fulfill an effector function in vivo (van den Hove et al, 1997). In addition, the low TNF-α mRNA levels in the $CD8^+$ TIL found in the present study indicate a possible anergy and low lytic capacity of this TIL subpopulation. This is in accordance with the finding that the composition of TIL depends on tumour grade, in as far as an increase in the percentage of $CD8^+$ cells positively correlates with the tumour grade and bad prognosis (Igarashi et al, 1992; Kowalczyk et al, 1997).

The anergy of the TIL may either be induced by the influence of other immunoregulatory cytokines such as IL-10 and IL-6 within the tumour microenvironment, or as a consequence of incomplete stimulation by tumour cells lacking co-stimulatory molecules (Chen et al, 1993). An elucidation of the exact function of these various cytokines may provide a better understanding of host–tumour interactions at the tumour site and may be of important clinical interest with respect to the potential use of TIL in adoptive cellular immunotherapy.

### REFERENCES

Alderson MR, Tough TW, Davis-Smith T, Braddy S, Falk B, Schooley KA, Goodwin RG, Smith CA, Ramsdell F and Lynch DH (1995) Fas ligand mediates activation-induced cell death in human T lymphocytes. *J Exp Med* **181**: 71–77

Angevin E, Kremer F, Gaudin C, Hercend T and Triebel F (1997) Analysis of T-cell immune response in renal cell carcinoma: polarization to type 1-like differentiation pattern, clonal T-cell expansion and tumour-specific cytotoxicity. *Int J Cancer* **72**: 431–440

Asselin-Paturel C, Echchakir H, Carayol G, Gay F, Opolon P, Grunenwald D, Chouaib S and Mami-Chouaib F (1998) Quantitative analysis of Th1, Th2 and TGF-beta l cytokine expression in tumour, TIL and PBL of non-small cell lung cancer patients. *Int J Cancer* **77**: 7–12

Belldegrun, A, Muul, LM and Rosenberg SA (1988) Interleukin 2 expanded tumour-infiltrating lymphocytes in human renal cell cancer: isolation, characterization, and antitumour activity. *Cancer Res* **48**: 206–214

Berke, G (1995) The CTL's kiss of death. *Cell* **81**: 9–12

Brouwenstijn N, Gaugler B, Krüse KM, van der Spek CW, Mulder A, Osanto S, van den Eynde BJ and Schrier PI (1996) Renal-cell carcinoma-specific lysis by cytotoxic T-lymphocyte clones isolated from peripheral blood lymphocytes and tumour-infiltrating lymphocytes. *Int J Cancer* **68**: 177–182

Brunner T, Mogil RJ, LaFace D, Yoo NJ, Mahboubi A, Echeverri F, Martin SJ, Force WR, Lynch DH, Ware CF and Green DR (1995) Cell-autonomous Fas (CD95)/Fas-ligand interaction mediates activation-induced apoptosis in T-cell hybridomas. *Nature* **373**: 441–444

Caignard A, Guillard M, Gaudin C, Escudier B, Triebel F and Dietrich P-Y (1996) *In situ* demonstration of renal-cell-carcinoma-specific T-cell clones. *Int J Cancer* **66**: 564–570

Chen L, Linsley PS and Hellstrom KE (1993) Costimulation of T cells for tumour immunity. *Immunol Today* **14**: 483–486

Dhein J, Walczak H, Bäumler C, Debatin K-M and Krammer PH (1995) Autocrine T-cell suicide mediated by APO-1/(Fas/CD95). *Nature* **373**: 438–441

Elsässer-Beile U, Wetterauer U, Schultze-Seemann W, Gallati H, Schulte Mönting J and von Kleist S (1996) Analysis of the immune reactivity of inflitrating and peripheral lymphocytes from patients with renal cell carcinoma by measuring cytokine secretion. *Cancer Immunol Immunother* **42**: 93–98

Elsässer-Beile U, Grussenmeyer T, Gierschner D, Schmoll B, Schultze-Seemann W, Wetterauer U and Schulte Mönting J (1999) Semiquantitative analysis of Th1 and Th2 cytokine expression in $CD3^+$-, $CD4^+$-, and $CD8^+$-renal cell carcinoma infiltrating lymphocytes. *Cancer Immunol Immunother* **48**: 204–208

Filguieria L, Zuber M, Merlo A, Caetano V, Schultz E, Harder F, Spagnoli, GC and Heberer M (1993) Cytokine gene transcription in renal cell carcinoma. *Br J Surg* **80**: 1322–1325

Finke JH, Rayman P, Edinger M, Tubbs RR, Stanley J, Klein E and Bukowski R (1992) Characterization of a human renal cell carcinoma specific cytotoxic CD8+ T cell line. *J Immunother* **11**: 1–11

Finke JH, Rayman P, Hart L, Alexander JP, Edinger MG, Tubbs RR, Klein E, Tuason L and Bukowski RM (1994) Characterization of tumour-infiltrating lymphocyte subsets from human renal cell carcinoma – specific reactivity defined by cytotoxicity, interferon-gamma secretion, and proliferation. *J Immunother* **15**: 91–104

Igarashi T, Murakami S, Takahashi H, Matsuzaki O and Shimazaki J (1992) Changes on distribution of CD4+/CD45RA- and CD8+/CD11-cells in tumour-infiltrating lymphocytes of renal cell carcinoma associated with tumour progression. *Eur Urol* **22**: 323–328

Ito N, Nakamura H, Tanaka Y and Ohgi S (1999) Lung carcinoma: analysis of T helper type 1 and 2 cells and T cytotoxic type 1 and 2 cells by intracellular cytokine detection with flow cytometry. *Cancer* **85**: 2359–2367

Kowalczyk D, Skorupski W, Kwias Z and Nowak J (1997) Flow cytometric analysis of tumour-infiltrating lymphocytes in patients with renal cell carcinoma. *Br J Urol* **80**: 543–784

Maeurer MJ, Martin DM, Castelli C, Elder E, Leder G, Storkus WJ and Lotze MT (1995) Host immune response in renal cell cancer – interleukin-4 (IL-4) and IL-10 mRNA are frequently detected in freshly collected tumour-infiltrating lymphocytes. *Cancer Immunol Immunother* **41**: 111–121

Martinez-Lorenzo MJ, Alava MA, Anel A, Pineiro A and Naval J (1996) Release of preformed Fas ligand in soluble form is the major factor for activation-induced death of Jurkat T cells. *Immunology* **89**: 511–517

Mitropoulos D, Kooi S, Rodriguez-Villanueva J and Platsoucas CD (1994) Characterization of fresh (uncultured) tumour-infiltrating lymphocytes (TIL) and TIL-derived T cell lines from patients with renal cell carcinoma. *Clin Exp Immunol* **97**: 321–327

Nagoshi M, Goedegebuure PS, Burger UL, Sadanaga N, Chang MP and Eberlein TJ (1998) Successful adoptive cellular immunotherapy is dependent on induction of a host immune response triggered by cytokine (IFN-gamma and granulocyte/macrophage colony-stimulating factor) producing donor tumour-infiltrating lymphocytes. *J Immunol* **160**: 334–344

Nagoshi M, Sadanaga N, Joo HG, Goedegebuure PS and Eberlein TJ (1999) tumour-specific cytokine release by donor T cells induces an effective host anti-tumour response through recruitment of host naive antigen presenting cells. *Int J Cancer* **80**: 308–314

Nakagomi H, Pisa P, Pisa EK, Yamamoto Y, Halapi E, Backlin K, Juhlin C and Kiessling R (1995) Lack of interleukin-2 (IL-2) expression and selective expression of IL-10 mRNA in human renal carcinoma. *Int J Cancer* **63**: 366–371

Ng SY, Gunning P, Eddy R, Ponte P, Leavitt J, Shows T and Kedes L (1985) Evolution of the functional human β-actin gene and its multi-pseudogene family: conservation of noncoding regions and chromosomal dispersion of pseudogenes. *Mol Cell Biol* **5**: 2720–2732

© 2000 Cancer Research Campaign

Olive C, Nicol D and Falk MC (1997) Characterisation of gamma delta T cells in renal cell carcinoma patients by polymerase chain reaction analysis of T cell receptor transcripts. *Cancer Immunol Immunother* **44**: 27–34

Ortegel JW, Staren ED, Faber LP, Warren WH and Braun DP (2000) Cytokine biosynthesis by tumour-infiltrating T lymphocytes from human non-small-cell lung carcinoma. *Cancer Immunol Immunother* **48**: 627–34

Pisa P, Halapi E, Pisa EK, Gerdin E, Hising C, Bucht A, Gerdin B and Kiessling R (1992) Selective expression of interleukin 10, interferon γ, and granulocyte-macrophage colony-stimulating factor in ovarian cancer biopsies. *Proc Natl Acad Sci USA* **89**: 7708–7712

Raff T, van der Giet M, Endemann D, Wiederholt T and Paul M (1997) Design and testing of beta-actin primers for RT-PCR that do not co-amplyfy processed pseudogenes. *Biotechniques* **23**: 456–460

Schendel DJ, Gansbacher B, Oberneder R, Krieglmair M, Hofstetter A, Riethmüller G and Segurado OG (1993) tumour-specific lysis of human renal cell carcinomas by tumour-infiltrating lymphocytes. I. HLA-A2-restricted recognition of autologous and allogeneic tumour lines. *J Immunol* **151**: 4209–4220

Tureci O, Sahin U, Vollmar E, Siemer S, Gottert E, Seitz G, Parkkila AK, Shah GN, Grubb JH, Pfreundschuh M and Sly WS (1998) Human carbonic anhydrase XII: cDNA cloning, expression, and chromosomal localization of a carbonic anhydrase gene that is overexpressed in some renal cell cancers. *Proc Natl Acad Sci USA* **95**: 7608–7613

van den Hove LE, van Gool SW, van Poppel H, Baert L, Coorevits L, van Damme B and Ceuppens JL (1997) Phenotype, cytokine production and cytolytic capacity of fresh (uncultured) tumour-infiltrating T lymphocytes in human renal cell carcinoma. *Clin Exp Immunol* **109**: 501–509

Wang Q, Redovan C, Tubbs R, Olencki T, Klein E, Kudoh S, Finke J and Bukowski RM (1995) Selective cytokine gene expression in renal cell carcinoma tumour cells and tumour-infiltrating lymphocytes. *Int J Cancer* **61**: 780–785

Whiteside TL, Miescher S, Hurlimann J, Moretta L and von Fliedner V (1986) Separation, phenotyping and limiting dilution analysis of T-lymphocytes infiltrating human solid tumours. *Int J Cancer* **37**: 803–811

Whiteside TL and Parmiani G (1994) tumour-infiltrating lymphocytes: their phenotype, functions and clinical use. *Cancer Immunol Immunother* **39**: 15–21

*© 2000 Cancer Research Campaign*
